# Dopamine Release Dynamics Change during Adolescence and after Voluntary Alcohol Intake

**DOI:** 10.1371/journal.pone.0096337

**Published:** 2014-05-01

**Authors:** Sara Palm, Ingrid Nylander

**Affiliations:** Neuropharmacology, Addiction & Behaviour, Department of Pharmaceutical Biosciences, Uppsala University, Uppsala, Sweden; University of Colorado, United States of America

## Abstract

Adolescence is associated with high impulsivity and risk taking, making adolescent individuals more inclined to use drugs. Early drug use is correlated to increased risk for substance use disorders later in life but the neurobiological basis is unclear. The brain undergoes extensive development during adolescence and disturbances at this time are hypothesized to contribute to increased vulnerability. The transition from controlled to compulsive drug use and addiction involve long-lasting changes in neural networks including a shift from the nucleus accumbens, mediating acute reinforcing effects, to recruitment of the dorsal striatum and habit formation. This study aimed to test the hypothesis of increased dopamine release after a pharmacological challenge in adolescent rats. Potassium-evoked dopamine release and uptake was investigated using chronoamperometric dopamine recordings in combination with a challenge by amphetamine in early and late adolescent rats and in adult rats. In addition, the consequences of voluntary alcohol intake during adolescence on these effects were investigated. The data show a gradual increase of evoked dopamine release with age, supporting previous studies suggesting that the pool of releasable dopamine increases with age. In contrast, a gradual decrease in evoked release with age was seen in response to amphetamine, supporting a proportionally larger storage pool of dopamine in younger animals. Dopamine measures after voluntary alcohol intake resulted in lower release amplitudes in response to potassium-chloride, indicating that alcohol affects the releasable pool of dopamine and this may have implications for vulnerability to addiction and other psychiatric diagnoses involving dopamine in the dorsal striatum.

## Introduction

Adolescence is associated with high impulsivity and risk-taking behavior, making adolescent individuals more inclined to use drugs [1]. Nicotine, alcohol or cannabis are likely tested before psychostimulants or opiates [2], [3] and early drug use is correlated to increased substance use disorders (SUD) later in life [4]–[6]. The neurobiology underlying this increased risk of SUD is unclear, but adolescence is a time of extensive brain development and disturbances of normal brain development by drugs of abuse is hypothesized to contribute to the increased vulnerability after adolescent drug use [7].

Drugs of abuse commonly act on the reward system and increase extracellular levels of dopamine in the nucleus accumbens acutely after intake [8]. However, the transition from initial drug use to compulsive use and addiction involve long-lasting changes in many of the neural networks [9] and one of them is hypothesized to involve a shift from the nucleus accumbens, mediating acute reinforcing effects, to recruitment of the dorsal striatum and habit formation [10]. The dopaminergic activity in the dorsal striatum could therefore also be a factor in the vulnerability of adolescent individuals.

Animal models are of great importance to our understanding of these mechanisms and the age window identified as adolescence in rodents is between postnatal day (PND) 28 and 50 [11]. Previous studies have shown that adolescent rats have a reduced basal rate of dopamine release, a reduced pool of readily releasable dopamine, but also a larger storage pool of dopamine compared to adults [12]. It has also been suggested that despite the reduced dopamine release under basal conditions, the adolescent individuals may be able to release more dopamine if stimulated by pharmacological challenges [13]. The first of objective of this study was therefore to test the hypothesis of increased dopamine release after a pharmacological challenge in adolescent animals. Dopamine release and uptake was investigated using chronoamperometric dopamine recordings in combination with a challenge by amphetamine in early and late adolescent, as well as adult, outbred Wistar rats.

The second objective of this study was to investigate the effect of environmental influence by voluntary alcohol intake during adolescence. The rationale behind this was that previous studies show that environmental factors during the adolescent period, such as intraperitoneally administered alcohol, increase basal extracellular levels of dopamine [14] while voluntary alcohol intake in alcohol-preferring P rats increase dopamine uptake, without affecting basal extracellular levels [15]. Discrepancies between these studies can be explained by a number of factors, such as route of administration, dose, rat strain and exact time period, but in both cases, adolescent alcohol intake affects the dopamine dynamics and this is well worth investigating further.

## Materials and Methods

### Ethics Statement

All animal experiments were performed under a protocol approved by the Uppsala Animal Ethical Committee and followed the guidelines of the Swedish Legislation on Animal Experimentation (Animal Welfare Act SFS1998∶56) and the European Communities Council Directive (86/609/EEC).

### Animals

Pregnant Wistar rats (RccHan: WI, Harlan Laboratories B.V., Horst, The Netherlands) arrived at the animal facility at gestation day 16. The animals arrived in batches over several weeks in order to accommodate the timing of the chronoamperometric recordings. The dams were single housed in macrolon cages (59 cm×38 cm×20 cm) with pellet food (Type R36; Lantmännen, Kimstad, Sweden) and tap water *ad libitum*. The cages contained wood-chip bedding and paper sheets (40×60 cm; Cellstoff, Papyrus) and were changed once a week by animal care personnel. The animal room was kept at constant temperature (22±1°C) and humidity (50±10%) on a regular 12 h light/dark cycle with lights on at 06∶00 am. All rooms had a masking background noise to minimize unexpected sounds that could disturb the animals.

**Figure 1:**
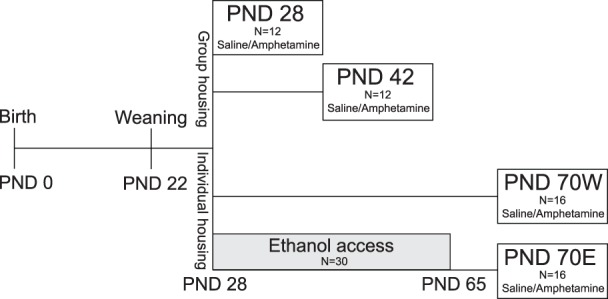
The experimental outline. E = ethanol-drinking, PND = postnatal day, W = water-drinking.

An overview of the experimental outline can be found in Figure 1. The litters that were born on the same day (postnatal day (PND) 0) were cross-fostered to include 6 males and 4 females in order to control for maternal shipping stress, maternal behavior and genetics. The pups were weaned on PND 22 and housed 3 per cage until PND 28 (±1 day) or PND 42 (±1 day) when chronoamperometric recordings were made. Only male pups were further used in this study. A group of thirty male rats were given voluntary binge-like access to 20% ethanol in a two-bottle free-choice paradigm from PND28 to PND65. The animals were given 24 hours access to ethanol for three consecutive days per week, i.e. Tuesday through Thursday for six weeks, a total of 18 sessions. For measures of ethanol intake, the bottles were weighed before and after each session and grams pure ethanol per kilogram body weight were calculated. Bottle positions were changed between sessions to avoid position preference. The ethanol-drinking animals were individually housed from PND 28 until PND 70. The animals with the highest cumulative ethanol intake (g/kg) were selected and electrochemical recordings were then made at PND 70 (±2 days). Age-matched water-drinking controls were also housed individually during the same period.

### Chronoamperometric Recordings of Dopamine *In* *vivo*

#### Materials

Inactin, Nafion 5% solution, dopamine hydrochloride, L-ascorbic acid, potassium chloride, sodium chloride, sodium phosphate, calcium chloride and d-amphetamine sulfate were obtained from Sigma-Aldrich, LLC (St Louis, MO, USA). Kerr sticky wax was obtained from DAB LAB AB (Upplands Väsby, Sweden). Carbon fibre microelectrodes (SF1A; 30 µm outer diameter×150 µm length) were purchased from Quanteon, LLC (Nicholasville, KY, USA), the reference electrode silver wire (200 µm, Teflon-insulated) was purchased from A-M Systems Inc. (Carlborg, WA, USA) and glass capillaries (0.58 mm inner diameter) for the micropipettes were purchased from World Precision Instruments Ltd (Stevenage, UK).

#### Surgery

Dopamine recordings were made at PND 28 (±1 day), PND 42 (±1 day) or PND 70 (±2 days). Surgery was performed immediately prior to the electrochemical recordings. A water-circulating heating pad (Gaymar Industries, Inc., Orchard Park, New York) was used to maintain body temperature. The animals were anesthetized with Inactin 125 mg/kg intraperitoneally (i.p.) and placed in a stereotaxic frame (Stoelting Co., Wood Dale, IL, USA). A hole in the skull was drilled overlying the recording site for the electrode, and another hole was drilled remote from the recording site for the placement of the Ag/AgCl reference electrode.

#### High-speed chronoamperometric recordings of dopamine release and uptake

High-speed chronoamperometric measurements (1 Hz sampling rate, 200 ms total) were performed using the FAST16-mkII recording system (Fast Analytical Sensing Technology, Quanteon, LLC, Nicholasville, KY, USA) according to a previously described procedure [16]. Carbon fibre microelectrodes (SF1A) were coated with three coats of Nafion with 5 min heating at 200°C before the first coating and after each coating [17]. The electrodes were then calibrated *in vitro* in 0.05 M phosphate buffered saline to determine selectivity, limit of detection (LOD) and slope before use *in vivo* [16]. The microelectrodes showed linear responses to serial additions of dopamine (2–6 µM), with an average correlation coefficient (R2) of 0.999±0.0003. The average selectivity for all electrodes used in this study was 14482±3005 µM for dopamine over ascorbic acid. The average LOD was 0.026±0.004 µM dopamine, and the average slope was −1.00±0.03 nA/µM dopamine. The average reduction/oxidation ratio measured during the reference peak responses of dopamine was 0.67±0.02, which is indicative of the detection of predominantly dopamine [17]. A silver wire was plated and used as the *in vivo* Ag/AgCl reference electrode [18].

#### In vivo experimental protocol

A micropipette (10–15 µm inner diameter) was filled with isotonic potassium chloride solution (120 mM KCl, 29 mM NaCl, 2.5 mM CaCl_2_·2H_2_O) (pH 7.2–7.4) using a pipette-filling needle (28G, World Precision Instruments, Aston, UK). The micropipette was affixed approximately 150–200 µm from the carbon fibre tip using sticky wax. The electrode was stereotactically placed in the dorsal striatum, AP: +1.0 mm, L: +3.0 mm from bregma, the incisor bar was adjusted according to age and weight [19], [20]. The electrode was initially placed dorsal (−3.0 mm) to the recording site, using a micromanipulator (Narishige International Ltd, London, UK) to lower it, and allowed to reach a stable baseline for about 45–60 min before being lowered to a depth of −4.0 mm from bregma. The electrode was then allowed another 5–10 min to stabilize at the recording site before the effect of a single injection of potassium chloride on dopamine release was determined. The potassium solution was locally applied using pressure ejection controlled by a PicoSpritzer III (Parker Hannifin Corporation, Pine Brook, NJ, USA) and the pressure (10–20 psi) and time (0.5–1.0 s) was adjusted to deliver 100 nl of the potassium solution, measured by a surgical microscope fitted with an eyepiece reticule [21].

**Figure 2:**
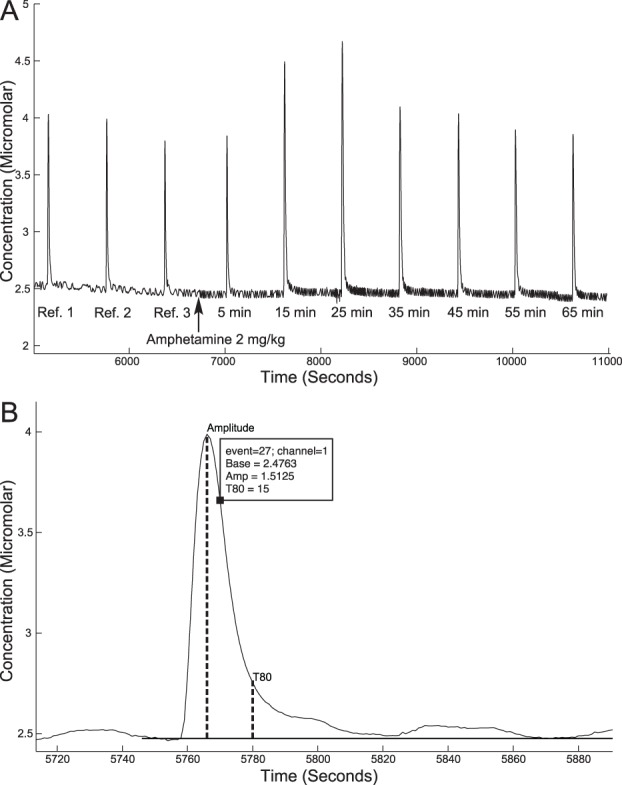
Representative traces. A) A representative trace of the oxidation current for a rat at postnatal day 28 receiving amphetamine and B) a close-up of the second reference peak for the same animal showing how amplitude and T80 were calculated. Amp  =  amplitude, Base  =  baseline, Ref  =  reference.

Potassium evoked release was used in combination with subcutaneous injections of amphetamine or saline. Three reference peaks similar in amplitude were produced, 10 min apart. Five min after the last reference peak, rats were given either 2 mg/kg amphetamine or the equivalent amount of saline (1 ml/kg) and after another 5 min release was again evoked every 10 min, producing peaks at 5, 15, 25, 35, 45, 55 and 65 min after the systemic injection, see Figure 2A for a representative trace. The dose of amphetamine was chosen based on behavioral effects in locomotion and self-administration studies [22]–[24].

#### Verification of electrode placement and exclusions

The electrodes were cut off and left in place after the finished experiment and the brains were frozen. The placement was verified by sectioning of the frozen brains. From the 12 animals at PND28, 1 was excluded due to wrongful placement, and 2 because of recording errors. For the 12 animals at PND 42, 1 animal was excluded due to wrongful placement. For the 16 animals at PND 70, 3 were excluded due to recording errors. For the 16 ethanol-drinking animals at PND 70, 2 were excluded due to recording errors. Recording errors include pipette clogging and electrical disturbances such as power cuts and disturbances of the general power supply to the recording unit.

#### Data analysis

The maximal amplitude of the evoked peaks and the time for the peak to decline to 80% of its amplitude (T80) were calculated using the FAST Analysis software version 4.4 (Quanteon, LLC, Nicholasville, KY, USA), see Figure 2B for a representative trace. The three reference peaks were averaged and the percentage of these peaks were calculated for the peaks following injection. For statistical analysis, repeated measures analysis of variance (ANOVA) was used to compare chronoamperometric data over time between ages or drinking groups and treatment (saline or amphetamine), followed by Fisher’s least significant difference (LSD) post-hoc test. For ethanol intake data, which was not normally distributed, Friedman ANOVA was used. Statistical analyses were performed using Statistica 10 (StatSoft Inc., Tulsa, OK, USA). Differences were considered statistically significant at p<0.05.

## Results

### Age-dependent Effects

**Figure 3:**
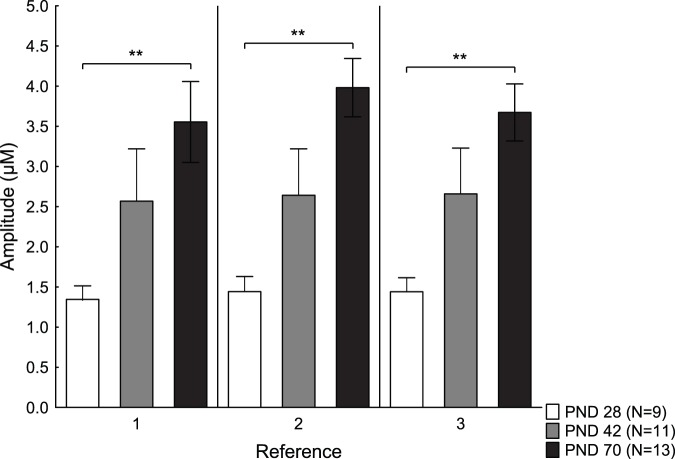
Reference peak amplitudes in different ages. Amplitudes (µM) (mean ± SEM) of the three reference peaks before treatment with either amphetamine or saline in the three age groups; postnatal day (PND) 28, 42 and 70. **p<0.01.

Differences in reference amplitudes between the age groups are shown in Figure 3. A repeated measures ANOVA comparing age and time, showed a main effect of age [F(2,22) = 5.81; p = 0.009], but no effect of time [F(2,44) = 1.43; p = 0.25] or any interaction effect between time and age [F(4,44) = 1.70; p = 0.17].

No effects of age [F(2,24) = 1.02; p = 0.38], time [F(2,48) = 0.94; p = 0.40] or time and age [F(4,48) = 0.22; p = 0.93] were found for the reference T80 values. The mean ± standard error of the mean (SEM) reference T80 values were 17.3±1.3 for the PND 28, 19.5±0.9 for the PND 42 and 20.5±1.0 for the PND70.

**Figure 4:**
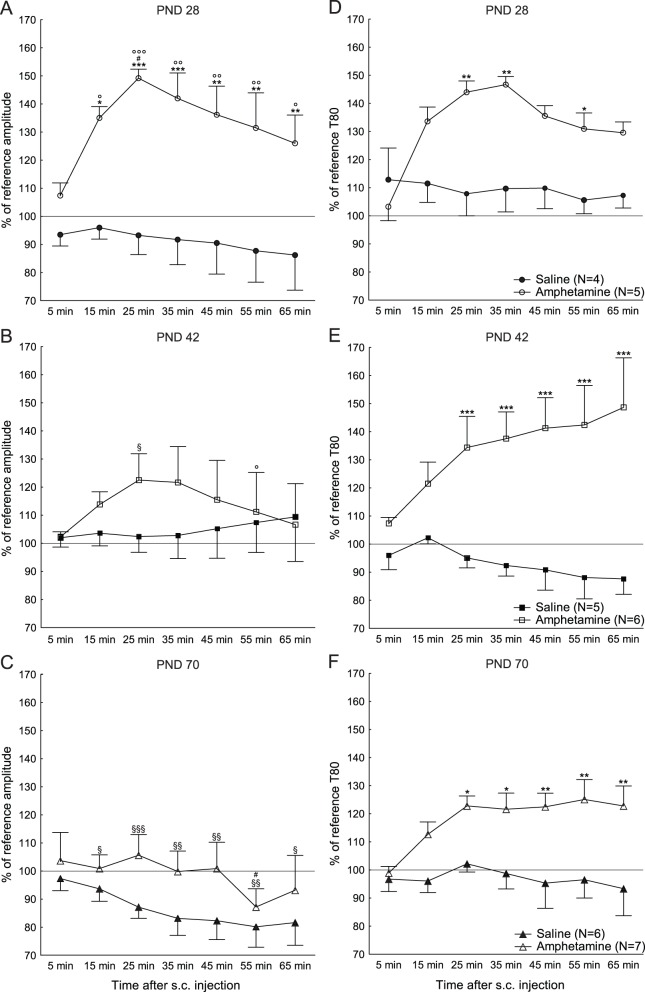
Amplitudes and T80 responses over time in different ages. Responses over time after subcutaneous (s.c.) injections of saline or amphetamine, as percent of reference values (mean ± SEM), for the amplitudes at A) postnatal day (PND) 28, B) PND 42 and C) PND 70, and for the T80 values at D) PND 28, E) PND 42 and F) PND 70. *p<0.05, **p<0.01, ***p<0.001 compared to saline controls, ^#^p<0.05 compared to the equivalent time-point at PND 42, °p<0.05, °°p<0.01, °°°p<0.001 compared to the equivalent time-point at PND 70, ^§^p<0.05, ^§§^p<0.01, ^§§§^p<0.001 compared to the equivalent time-point at PND 28.

Differences between the age groups in amplitude response to amphetamine are shown in Figure 4A–C. Amphetamine treatment resulted in main effects of age [F(2,26) = 3.95; p = 0.03], treatment [F(1,26) = 10.77; p = 0.003] and time [F(6,156) = 3.32; p = 0.004], and interaction effects between time and age [F(12,156) = 2.23; p = 0.01], time and treatment [F(6,156) = 4.20; p<0.001], but no interaction between age and treatment [F(2,26) = 2.37; p = 0.11] or time, age and treatment [F(12,156) = 0.77; p = 0.68].

The T80 response to amphetamine is shown in Figure 4D–E. There was no main effect of age [F(2,25) = 1.87; p = 0.17], but there were effects of treatment [F(1,25) = 26.52; p<0.001], time [F(6,150) = 7.70; p<0.001] and an interaction effect of time and treatment [F(6,150) = 12.29; p<0.001]. There was no interaction between age and treatment [F(2,25) = 1.29; p = 0.29], time and age [F(12,150) = 0.66; p = 0.78] and a trend towards an interaction between time, age and treatment [F(12,150) = 1.60; p = 0.098].

### Voluntary Adolescent Alcohol Intake

Ethanol intake data for the 14 rats that were used in the chronoamperometric recordings are shown in Table 1. A Friedman ANOVA showed no significant differences in intake over time, although there was a trend [χ*^2^* = 9.80; p = 0.08] towards differences driven by the intake during the second week (PND 35–37), which was slightly higher than the following weeks. A Friedman ANOVA of the preference showed an increase over time [χ*^2^* = 19.7; p = 0.001], mainly as a result from increases over the first three weeks, see Table 1.

**Table 1:** The median, minimum and maximum alcohol intake (g/kg/24 h) and preference (%) for the six weeks of alcohol access and the median, minimum and maximum cumulative intake (g) after the 18 sessions.

Week	Median intake	Min	Max	Median preference	Min	Max
1	3.5	3.1	7.5	11.9	9.6	19.1
2	4.1	2.6	8.9	15.2*	8.9	31.0
3	3.6	2.2	6.5	16.9*	9.5	35.3
4	3.5	1.8	7.0	17.5	9.3	35.5
5	3.3	1.9	6.0	19.4	9.8	36.9
6	3.6	1.7	5.6	22.9	10.4	34.5
Cumulative	12.5	9.0	22.0			

*p<0.05 compared to the previous week.

**Figure 5:**
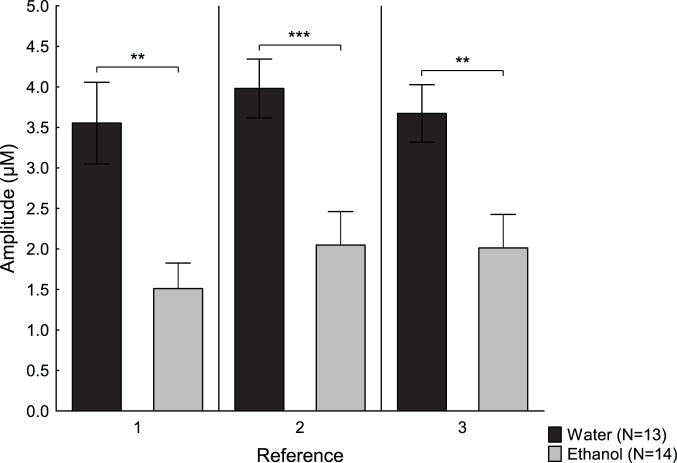
Reference peak amplitudes in water- or ethanol-drinking animals. Amplitudes (µM) (mean ± SEM) of the three reference peaks before treatment with either amphetamine or saline in the water- and ethanol-drinking groups. **p<0.01, ***p<0.001.

Differences in reference amplitudes between the ethanol- and water-drinking groups are shown in Figure 5. A repeated measures ANOVA comparing drinking group and time, showed a main effect of drinking group [F(1,17) = 16.22; p<0.001], but no effect of time [F(2,34) = 1.76; p = 0.19] or any interaction effect between time and drinking group [F(4,44) = 1.32; p = 0.28].

No effects of drinking group [F(1,18) = 0.04; p = 0.85], time [F(2,36) = 1.96; p = 0.16] or time and drinking group [F(2,36) = 0.22; p = 0.81] were found for the reference T80 values. The mean ± SEM reference T80 values were 20.5±1.0 for the water-drinking rats, and 19.1±1.3 for the ethanol-drinking rats.

**Figure 6:**
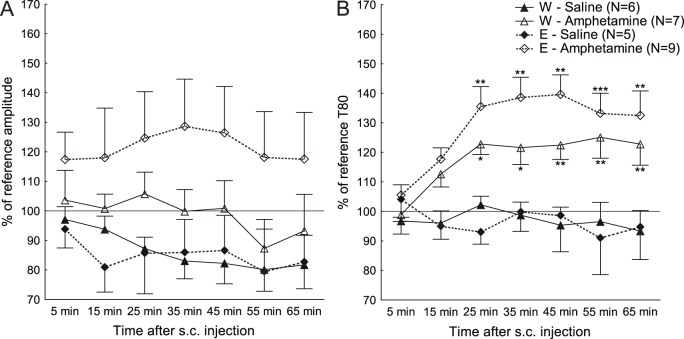
Amplitudes and T80 responses over time in water- or ethanol-drinking animals. Responses over time after subcutaneous (s.c.) injections of saline or amphetamine, as percent of reference values (mean ± SEM), for the A) amplitudes and B) T80 values in the water (W)- or ethanol (E)-drinking groups. *p<0.05, **p<0.01, ***p<0.001 compared to saline controls.

The response to amphetamine in the ethanol- and water-drinking groups is shown in Figure 6. For the amplitudes, as shown in Figure 6A, there was a trend to an effect of treatment [F(1,19) = 3.01; p = 0.099] and there was a main effect of time [F(6,114) = 2.30; p = 0.04], but no effect of drinking group [F(1,19) = 0.39; p = 0.54] or any interaction effects between treatment and drinking group [F(1,19) = 0.83; p = 0.37] or time and treatment [F(6,114) = 1.13; p = 0.35], time and drinking group [F(6,114) = 0.44; p = 0.85] or time, treatment and drinking group [F(6,114) = 0.27; p = 0.95].

For the T80 values, Figure 6B, there was a main effect of treatment [F(1,19) = 17.35; p<0.001] and time [F(6,114) = 2.42; p = 0.03], and an interaction effect between time and treatment [F(6,114) = 10.28; p<0.001]. There was no effect of drinking group [F(1,19) = 0.33; p = 0.57], or any interaction effect between treatment and drinking group [F(1,19) = 0.76; p = 0.40], time and drinking group [F(6,114) = 1.66; p = 0.14], or time, treatment and drinking group [F(6,114) = 1.75; p = 0.12].

## Discussion

Age-dependent effects on dopamine release and uptake was investigated under basal conditions and in response to amphetamine in early and late adolescent, as well as adult, rats. The impact of alcohol drinking during adolescence was also examined and is, to our knowledge, the first study investigating release and uptake in voluntary drinking adolescent rats with a chronoamperometric technique.

### Age-dependent Effects

The age-dependent differences in reference amplitudes are in agreement with a previous study using voltammetry in combination with electrical stimulation, which showed that adult rats released more dopamine upon stimulation than young rats [12]. The time-point for adolescence used by Stamford (1989) was approximately PND 30, but since then studies have shown that around PND 40–45 there are peaks in basal extracellular levels of dopamine [25]–[27] and dopamine receptor D_2_ density [28], while tyrosine hydroxylase levels are lower than both early adolescence and adulthood [29]. The current study therefore included two time-points during adolescence, PND 28 and PND 42, equivalent to early and late adolescence [11]. Amplitudes in late adolescent animals were intermediate to amplitudes in early adolescence and adulthood, indicating that development from adolescence into adulthood involved a gradual increase in the release capacity of dopamine in response to potassium chloride in the dorsal striatum. This is consistent with reports of increased extracellular levels of dopamine in the nucleus accumbens in adulthood compared to adolescence [30], [31]. As previously mentioned, some studies also show peak levels at PND 45 [25]–[27] and they can be reconciled with the current study through reports of increased firing rates around the same PND [32], [33]. The current study did not measure basal extracellular levels and it is possible that an increased firing rate results in increased basal levels without any peak in potassium-induced release. Furthermore, one of the studies showing potassium-induced extracellular levels in the nucleus accumbens to peak around PND 42 [25] contrasts to data from the dorsal striatum, from Stamford (1989) and the current study, which indicate regional differences.

The uptake measure, T80, did not reveal any differences between the ages in the current study, whereas Stamford (1989) found that the rate of uptake was higher in adult rats. This can be due to methodological differences in the measure of uptake; T80 includes both the linear and the curvilinear part of the curve, whereas Stamford used the linear part of the curve [34]. The concentrations reached in this study are only a tenth of the concentrations in the previous study and V_max_ should therefore not be reached. Using the linear part of the peak curve to calculate uptake rate under these conditions will only produce uptake rates dependent on amplitudes [35]. T80 was chosen because it also takes into account the curvilinear part of the curve, where dopamine concentrations are lower and is more sensitive to dopamine uptake blockers [35], [36]. Naturally, T80 is also dependent on the amplitude, but as can be seen in this study, differences in amplitude do not automatically result in differences in T80, suggesting that the ratio of uptake to release is shifted towards uptake in the younger animals. Supporting the current findings is a study that used quantitative microdialysis and found no differences in the extraction fraction, an indirect measure of uptake rate, in the nucleus accumbens of rats at PND 35, 45 and 60 [26].

The greater potassium-evoked release in adulthood could be due to a larger releasable pool of dopamine [12] and a number of factors could be involved, such as age-dependent differences in dopamine synthesis by tyrosine hydroxylase [29], [37], vesicular monoamine transporter-2 (VMAT-2)-containing vesicles [38], and kinetics of the VMAT-2 [39], as well as D2 receptor pruning [28] and function [40]. These factors could also help explain the increased amplitudes seen after amphetamine in early adolescent animals. Again, the current data are consistent with data showing a larger increase in dopamine release in young compared to adult animals in response to nomifensine [12] indicating that early adolescent rats have a proportionally larger storage pool, which can be released upon stimulation by psychoactive substances. This is further supported by data showing a larger increase in stimulated extracellular dopamine after amphetamine in adolescent animals [22]. However, there are microdialysis studies showing lower extracellular levels of dopamine after amphetamine in adolescents compared to adults [30], [37], which again underline that a possibility of increase in stimulated release does not necessarily mean an increase in extracellular levels and that different techniques can add complementing information.

No age-dependent effects on T80 after amphetamine was found, which indicates that amphetamine exerts similar effects on dopamine uptake in all ages. This is again supported by the results from Stamford (1989), showing no differences in the degree of uptake blockade after nomifensine between age groups. There are also studies suggesting that age-related differences in dopamine transporter structure and function are related to the cocaine-binding site on the transporter, but not the amphetamine-binding site [22] which could indicate that age-dependent effects of amphetamine on uptake do not exist. However, there was a trend towards an interaction between time, age and treatment, suggesting that they responded differently over time to amphetamine depending on age. Further studies investigating the uptake by applying exogenous dopamine could also help separate amplitude-dependent uptake from transporter function [41]–[43]. Studies in awake rats would also be of importance, as the current study was done in anesthetized animals. The anesthesia used was the barbiturate thiobutabarbital (Inactin), a positive allosteric modulator of gamma-aminobutyric acid (GABA) A receptors, which produces a prolonged and stable anesthesia in rats [44]. GABA may exert different effects depending on age and alcohol-drinking history [45] and therefore, the anesthesia could interact with age or treatment and produce confounding effects. However, pentobarbital, another barbiturate, has been shown to have little effect on dopamine levels in the striatum [46]. Furthermore, in the current study, release was induced using potassium chloride and did not rely on spontaneous events, which should decrease the importance of GABAergic tone on release. As for the dopamine uptake there are reports that barbiturates may affect dopamine uptake specifically [47], but whether there could also be an interaction with age or treatment is unclear.

### Voluntary Adolescent Alcohol Intake

Voluntary adolescent alcohol intake for six weeks resulted in lower reference amplitudes compared to water-drinking controls. The amplitudes were similar to those seen in early adolescent rats. Since the effects were seen in amplitudes and not uptake time, it is conceivable that alcohol affects factors controlling the releasable pool of dopamine rather than the dopamine transporter and there are data supporting unaffected uptake after adolescent alcohol [14]. There are also microdialysis data showing increased extracellular levels of dopamine after adolescent exposure to intraperitoneal injections of alcohol [14], [27], [48], and this is somewhat contradictory to the current findings of decreased releasable dopamine. As mentioned earlier, increased firing rates may be a way of reconciling the microdialysis data with the current data, but there are no studies to support this. Furthermore, there are studies showing that the mode of alcohol exposure, i.e. voluntary or forced, may have different effects on the neurobiology [49].

When treated with amphetamine, there were no significant differences between the alcohol- and water-drinking groups in amplitudes or T80. However, there was a trend towards an effect on amplitudes, due to the increase displayed by the alcohol-drinking group. There is also more variation in the response to amphetamine in the alcohol-drinking group, which could be due to a variation in alcohol intake, although this variation is not correlated to the response (data not shown). This also points to a limitation with this study, namely that blood alcohol levels were not measured. The study was based on undisturbed access for 24 h and for blood alcohol levels to be measured the access would have had to be limited and the stress involved in blood sampling would have risked to disturb the intake patterns of the animals. Thus, correlations between response and individual blood alcohol levels cannot be ruled out. However, the intake data presented in this study is similar to other studies, showing neurobiological effects of alcohol, using Wistar rats in similar ages or intake paradigms[50]–[52]. This suggests that not only individuals prone to high intake, but also modest drinkers from a cross-section of a general population, risk changes in neurobiology after voluntary adolescent alcohol intake.

No differences in uptake time after amphetamine suggests that adolescent alcohol has no effect on dopamine transporter function in response to amphetamine, but would also benefit from investigation by application of exogenous dopamine [41]–[43].

Furthermore, two interesting observations were made. Firstly, the reference amplitudes after alcohol intake are similar to those seen in animals at the beginning of the alcohol intake period, i.e. PND 28. Secondly, the size of the increase in amplitudes after amphetamine in the alcohol-drinking animals is similar to the late adolescent rats, i.e. PND 42. Whether these findings relate to altered development of the releasable pool and the storage pool of dopamine in the neurons remains to be elucidated. The current study did not include a group of adult alcohol-drinking rats so conclusions about the possibility of age-specific effects cannot be drawn. However, indications of age-specific effects can be found in the discrepancies between studies of adolescent alcohol-exposed rats that show unaffected dopamine uptake [14] and studies of adult alcohol-exposed rats and monkeys that show increased uptake, but no effects on evoked dopamine overflow [53], [54]. For future studies, it would therefore be of great interest to investigate alcohol exposure and the mechanisms behind its effect in different ages. Further investigations into factors such as tyrosine hydroxylase, dopamine receptor density and function, and vesicular monoamine transporter could help shed some light on possible age-specific alcohol effects on the releasable pool and the storage pool of dopamine. To our knowledge, these factors have not been investigated after adolescent alcohol.

## Conclusion

The data show a gradual increase of evoked dopamine overflow with age, supporting previous studies suggesting that the pool of releasable dopamine increases with age. In contrast, a gradual decrease in evoked overflow with age was seen in response to amphetamine, supporting a proportionally larger storage pool of dopamine in younger animals, making them potentially more sensitive to dopamine-releasing drugs. Adolescent alcohol intake resulted in overflow lower than in water-drinking controls., indicating that alcohol affects the releasable pool of dopamine and this may have implications for vulnerability to addiction and other psychiatric diagnoses involving the dopamine system in the dorsal striatum.
